# La tachycardie atriale mal tolérée du nouveau-né: à propos d’un cas

**DOI:** 10.11604/pamj.2019.34.176.19883

**Published:** 2019-12-04

**Authors:** Désiré Alain Affangla, Thérèse Yandé Sarr, Malick Ndiaye, Abib Laye Diedhiou, Franck D’Almeida, Djibril Marie Ba, Mohamed Leye, Adamson Phiri, Georges Antoine Bazolo Ba Ngouala, Adama Kane

**Affiliations:** 1 UFR des Sciences de la Santé, Université de Thiès, Sénégal; 2 Hôpital Saint Jean de Dieu, Thiès, Sénégal; 3 Centre Hospitalier Régional de Louga, Sénégal; 4 UFR Sciences de la Santé, Université Gaston Berger de Saint Louis, Sénégal

**Keywords:** Trouble du rythme, tachycardie supra ventriculaire, nouveau-né, Cardiac rhythm disorder, supraventricular tachycardia, newborn

## Abstract

Les troubles du rythme cardiaque mal tolérés du nouveau-né sont une véritable urgence nécessitant une prise en charge rapide et adéquate. Nous rapportons le cas d'un nouveau-né de 15 jours présentant une tachycardie atriale en défaillance cardiaque aiguë ayant nécessité une cardioversion électrique.

## Abstract (en)

Poorly tolerated cardiac rhythm disorders in the newborn are a real emergency requiring early and adequate management. We here report the case of 15-day old newborn with atrial tachycardia associated with acute heart failure requiring electrical cardioversion.

## Introduction

Les troubles du rythme cardiaque mal tolérés du nouveau-né constituent une véritable urgence diagnostique et thérapeutique. Nous rapportons un cas de tachycardie atriale mal tolérée chez un nouveau-né.

## Patient et observation

Il s'agissait d'un nouveau-né de sexe masculin âgé de 15 jours reçu pour détresse respiratoire. La dyspnée évoluait depuis une semaine avant son admission et d'aggravation progressive. La respiration était polypnéique avec une fréquence respiratoire à 51 cycles/minute, une SpO_2_ à l'air libre = 80%, une tachycardie régulière extrême avec une fréquence cardiaque à 250 battements /minute. On ne notait ni fièvre, ni diarrhée ni vomissement. La température était à 36,5°C, le poids = 4kg, la taille = 55cm, la surface corporelle = 0,24m^2^. Les muqueuses étaient bien colorées sans cyanose. L'examen retrouvait des signes de lutte avec un score de Silverman =7/10, des poumons libres, une tachycardie régulière sans souffle et une hépatomégalie. Par ailleurs la réactivité et la tonicité étaient estimées moyennes. Il était né d'une mère âgée de 26 ans, 4 gestes 4 pares et nourri par allaitement maternel exclusif. La grossesse a été bien suivie avec 4 consultations prénatales. L'accouchement à terme était eutocique par voie basse, sans notion de réanimation avec un score APGAR = 10 à la première minute.

**Figure 1:**
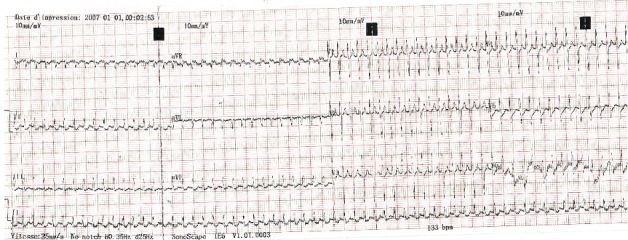
Tachycardie atriale à 300/min

Sur le plan biologique, on notait à la numération formule sanguine une hyperleucocytose à prédominance neutrophile avec des globules blancs à 11470 éléments/mm^3^, une CRP élevée à 12UI, un taux d'hémoglobine à 16,2 g/dl, des plaquettes à 232000 éléments/mm^3^. La fonction rénale était normale avec une créatininémie à 08mg /l. L'ionogramme sanguin notait une hyperkaliémie à 6,2 mEq/ l. L'ECG 12 dérivations à l'admission inscrivait une tachycardie régulière à QRS fins à 300 battements par minutes (Figure 1). On observe en D3 une activité positive juste après les QRS qui nous permet d'exclure une tachycardie jonctionnelle. Une manœuvre vagale par compression des globes oculaires était négative. Une échographie cardiaque rapide gênée par la tachycardie extrême paraissait normale. Le diagnostic d'une tachycardie atriale mal tolérée en insuffisance cardiaque aiguë associée à probable une infection néonatale tardive a été retenue.

**Figure 2:**
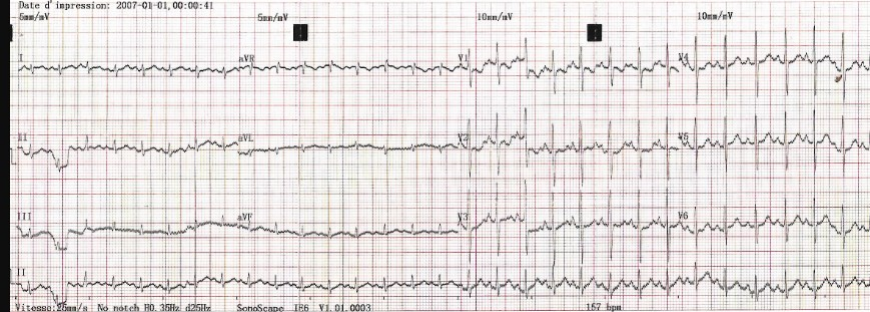
Tachycardie sinusale à 150/min

**Figure 3:**
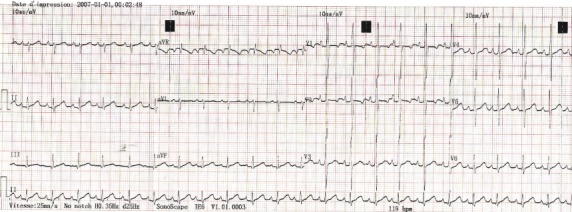
Rythme sinusal à 115/min

Dès lors une oxygénothérapie à 3l/min à la lunette, une dose de charge d'Amiodarone (500mg/m^2^) per os par sonde naso-gastrique et une antibiothérapie par la Céfotaxime à 100mg/Kg par voie intraveineuse et ont été administrées suivies dans les minutes d'un choc électrique externe (CEE) à 05 joules permettant un retour en rythme sinusal à 150/min avec une déviation axiale droite de QRS à 120° (Figure 2). L'évolution était marquée dans les heures qui suivent par une nette amélioration caractérisée par une régression complète des signes de l'insuffisance cardiaque. L'échographie Doppler cardiaque refaite 2 jours après, dans de meilleures conditions montrait un cœur morphologiquement normal avec une excellente fonction systolique ventriculaire gauche (FEVG = 67%). La suite du traitement a constitué à une poursuite de la Céfotaxime pendant 08 jours et de l'Amiodarone à une dose d'entretien (200mg/m^2^) pendant 30 jours. Un ECG effectué 15 jours après la réduction du trouble du rythme cardiaque par cardioversion montre le maintien en rythme sinusal régulier à 115/min (Figure 3), une kaliémie normale à 4,4mEq/l.

## Discussion

La prise en charge des troubles du rythme cardiaque du nouveau-né dépend d'une part de la tolérance hémodynamique et d'autre part du diagnostic précis basé sur au moins l'analyse d'un ECG 12 dérivations [1]. Les tachycardies supraventriculaires en sont une des fréquentes présentations se révélant au cours des premières semaines de vie par une insuffisance cardiaque voire un collapsus cardiovasculaire dans 60% des cas [2,3]. Chez notre patient, le mode de révélation du trouble du rythme était en effet une insuffisance cardiaque aiguë qui engageait sont pronostic vital immédiat. Au plan biologique on notait une hyperkaliémie réversible traduisant une probable acidose métabolique. Une gazométrie artérielle non disponible aurait confirmé avec certitude le diagnostic de l'acidose. Il n'y avait pas de retentissement viscéral rénal et ventriculaire gauche chez notre patient. La créatininémie et la fonction systolique ventriculaire gauche étaient normales. Un risque évolutif immédiat et à court terme est représenté par l'insuffisance rénale et la cardiomyopathie rythmique [1,4]. Par ailleurs on notait des stigmates biologiques d'une infection néonatale probable caractérisée par l'élévation de la CRP et l'hyperleucocytose modérée. L'infection chez notre patient serait un diagnostic d'association car il n'y aurait pas de lien de causalité entre l'infection et le trouble du rythme cardiaque. L'origine supraventriculaire du trouble du rythme cardiaque chez notre patient est retenue sur la base du caractère fin des complexes rapides QRS, inférieur à 8/100 sec (Figure 1). La relation entre les ondes P' et les QRS n'a pu être clairement établie malgré une manouvre vagale. L'activité positive juste après les QRS en D3 nous permet cependant d'exclure une tachycardie jonctionnelle. Une injection intraveineuse de stryadine, qui n'était pas disponible est une alternative efficace recommandée pour le diagnostic exacte des tachycardies supraventriculaires du nouveau-né et du nourrisson [1,2,4]. Cependant devant la mauvaise tolérance hémodynamique et l'absence d'onde delta et d'un PR court sur l'ECG en rythme sinusal (Figures 2 et 3) le diagnostic de la tachycarde atriale est le plus probable. Le choc électrique externe est un geste salvateur devant un trouble du rythme cardiaque mal tolérée [1,2,4]. Néanmoins, il doit être effectué en toute sécurité par un matériel adapté pour nouveau-né permettant de délivrer la bonne dose d'énergie électrique notamment de 1 à 2 Joules /Kg [1] voire 4 Joules/joules/Kg [5]. Son efficacité peut être améliorée par une imprégnation préalable par l'Amiodarone qui va être poursuivi afin de réduire le risque de récidive du trouble du rythme qui est une modalité évolutive [1,2]. La durée du traitement d'entretien proposée est de 1 à 3 mois [2,6]. Le traitement par Amiodarone est interrompu à 30 jours chez notre patient. Une surveillance mensuelle régulière sera effectuée jusqu'à 3 mois.

## Conclusion

Le choc électrique externe est le traitement de première intention de la tachycardie atriale mal tolérée du nouveau-né. L'Amiodarone per os est l'anti arythmique de choix.

## Conflits d’intérêts

Les auteurs ne déclarent aucun conflit d’intérêts.
